# Biomimetic Electrospun Self-Assembling Peptide Scaffolds for Neural Stem Cell Transplantation in Neural Tissue Engineering

**DOI:** 10.3390/pharmaceutics15092261

**Published:** 2023-08-31

**Authors:** Mahdi Forouharshad, Andrea Raspa, Amanda Marchini, Maria Gessica Ciulla, Alice Magnoni, Fabrizio Gelain

**Affiliations:** 1 Institute for Stem-Cell Biology, Regenerative Medicine and Innovative Therapies, IRCCS Casa Sollievo della Sofferenza, 71013 San Giovanni Rotondo, Italy; 2 Center for Nanomedicine and Tissue Engineering (CNTE), ASST Grande Ospedale Metropolitano Niguarda, 20162 Milan, Italy; 3 Department of Biotechnology and Biosciences, University of Milan-Bicocca, 20125 Milan, Italy

**Keywords:** self-assembling peptides, electrospinning, regenerative medicine, spinal cord injury, secondary structures, 2D/3D scaffolds

## Abstract

Spinal cord regeneration using stem cell transplantation is a promising strategy for regenerative therapy. Stem cells transplanted onto scaffolds that can mimic natural extracellular matrix (ECM) have the potential to significantly improve outcomes. In this study, we strived to develop a cell carrier by culturing neural stem cells (NSCs) onto electrospun 2D and 3D constructs made up of specific crosslinked functionalized self-assembling peptides (SAPs) featuring enhanced biomimetic and biomechanical properties. Morphology, architecture, and secondary structures of electrospun scaffolds in the solid-state and electrospinning solution were studied step by step. Morphological studies showed the benefit of mixed peptides and surfactants as additives to form thinner, uniform, and defect-free fibers. It has been observed that β-sheet conformation as evidence of self-assembling has been predominant throughout the process except for the electrospinning solution. In vitro NSCs seeded on electrospun SAP scaffolds in 2D and 3D conditions displayed desirable proliferation, viability, and differentiation in comparison to the gold standard. In vivo biocompatibility assay confirmed the permissibility of implanted fibrous channels by foreign body reaction. The results of this study demonstrated that fibrous 2D/3D electrospun SAP scaffolds, when shaped as micro-channels, can be suitable to support NSC transplantation for regeneration following spinal cord injury.

## 1. Introduction

Owing to the rapid increase in demand for prostheses all over the world during the past decade, researchers in the tissue engineering field are driven by the necessity to create novel substitutes to replace damaged tissues or organs.

ECM, as a non-cellular structure, is a primary factor required in tissue engineering since it mechanically supports and anchors cells and regulates and determines cell dynamics and behaviors such as cell survival, proliferation, differentiation, and migration. The ECM is also involved in tissue growth, regeneration, and healing. Since the key to the success of ECM lies in its interactions with the cells, its components, architecture, and fibrillar structure play significant roles in biomimetic techniques [1,2,3,4].

The spinal cord belongs to the central nervous system and is unable to repair itself effectively due to its limited capacity for spontaneous regeneration and the onset of the harmful inflammatory cascades taking place at the site of injury. Thus, spinal cord injury with loss of motor and sensory functions causes permanent or semi-permanent neurological impairments [5,6]. Although there is no fundamental treatment available to replace neural tissue and restore certain functions, the rapidly burgeoning knowledge is striving to find effective therapies to help people with spinal cord injuries obtain a degree of health to guarantee an independent life [7,8,9]. Spinal cord regeneration using stem cell transplantation has demonstrated a promising regenerative therapy by promoting angiogenesis and neurogenesis [4,5]. Although some studies have shown promising cell survival, integration with host tissue, and new synapses that lead to improved recovery after spinal cord injury, some limitations still exist, such as retention, poor engraftment, low neural plasticity, uncontrolled differentiation of transplanted stem cells, oxidative stress, lack of growth factors, and limited vascularization [4,10,11,12]. Bioactive biomaterial-based scaffolds have been shown to impact many of these aforementioned aspects to improve the outcome of stem cells pre/post-transplantation by biomimicking the tissue microenvironments [4,13]. Although injectable biomaterials have been utilized successfully for stem cell transplantation, implantable biomaterials-based scaffolds have been shown to improve cell survival due to their mimicry of the complex in vivo cellular microenvironments [14,15,16,17]. Scaffold-transplanted stem cells formed more complex tissue architecture, improved cell retention, and better integrated with host tissue by improving cell migration [18]. On the other hand, since the environmental cues can fundamentally change the fate and functions of cells by tuning cell-cell, and cell-scaffold interactions, the architecture of the scaffold plays a vital role in cell behavior and morphology. Despite some advantages of conventional 2D cell models, including simplicity, low cost, and many available functional assays, more accuracy to mimic conditions in vivo makes 3D cell cultures physiologically more relevant and predictive than 2D cultures for cell modeling and transplantation [19,20,21].

To achieve this aim, high-porosity and bioactive fibrous 3D scaffolds are needed to successfully provide trophic factors and physical cues to transplanted cells required for cell-based therapies for neural regeneration [22].

Engineered SAPs hydrogel, made up of various backbone amino acid sequences and building bioactive motifs, has demonstrated biocompatibility, moderate immunomodulating activity, bio-absorbability, and non-toxic byproducts. In addition, the self-assembling conformation and nano-fibrillar architecture of SAPs, useful to mimic ECM, unique features of primary and secondary structures, biofunctionalities, and versatility by tailoring backbone and functional motifs, make them favorable for a variety of cell lines [23,24]. Despite the privileges and the great potential of self-assembling peptides as ECM “substitutes”, due to weak interaction (H-bonding, hydrophobic) involved in the self-assembling, they are still considered soft hydrogels that require to be bolstered via crosslinking covalent reactions to become suitable for the regeneration of medium-to-hard tissues [13,25,26].

Since the casting of SAP hydrogels in nano–micro scales has some drawbacks that cannot meet demands for tissue engineering, other fabrication techniques have been considered seriously [27]. To this aim, merging self-assembling peptides, a synthetic and biomimetic biomaterial with random nanofibrous orientation, with electrospinning, an applicable and versatile technique, as a novel approach to fabricating continuous nanofibers and scaffolds with specific 3D architectures, can lead the race to mimic the sophisticated fibrous network structure and bioactivity of ECM [26,27,28,29,30,31,32]. It is worth underlining that although morphological, mechanical, and biological studies of electrospun SAPs have confirmed their desirability as a scaffold in tissue engineering, it still needs more studies on the phase structures, secondary structures, and self-assembling conformation, i.e., most important factors influencing their biological activities and mechanical properties.

Amino acid sequences, peptide concentrations, pH, temperature, and solvent composition (in accordance with the solution or solid form) can regulate the secondary structure of peptides such as β-sheet and α-helix [23]. These conditions can significantly influence the dynamic peptide structures as well as the self-assembly process. The electrospinning process involves opposite electrostatic fields, governed by the electrohydrodynamic phenomena, to turn the charged solution into the rearranged solid nano/microfibrous construction: for their turn, these phenomena can also influence the SAP structure and conformation [27,30].

Given the efficient parameters on the conformational structure of peptides before and after the crosslinking and electrospinning process, monitoring of the secondary structure of SAPs in each step can lead to yielding an efficient scaffold with robust mechanical properties as a cell carrier.

In this present study, we assessed scaffold biocompatibility by culturing hNSCs onto electrospun 2D and 3D constructs made up of specific SAPs named biofunctionalized FAQ(LDLK)_3_ (Ac-FAQRVPP-GGG-(LDLK)_3_-CONH_2_) and HYDROSAP including functionalized linear and branched SAPs. Such SAPs are prone to this study because, in our previous work, they have demonstrated significant biomimetic and biomechanical properties [33,34]. SAPs were synthesized and crosslinked with Genipin, a natural-derived compound whose biocompatibility and lower cytotoxicity as compared with alternative crosslinkers have been confirmed [35,36]. Afterward, they were electrospun as 2D and 3D scaffolds, while the secondary structures in the solid-state and electrospinning solution were monitored step by step by the Fourier transform infrared (FTIR) method to interpret amid I and II regions, which are known as the most prominent vibrational bands of the peptide backbone, and to discern the secondary structure of SAPs. Three mathematical resolution enhancement methods—Fourier self-deconvolution, second derivative analysis, and band curve-fitting—were used to study individual secondary structures within the highly complex amide I band, which is caused by the overlap of several peaks. These techniques allow the quantitative estimation of peptides’ secondary structures [37]. The morphology of electrospun SAP fibers and the architecture of 2D laminas and 3D channels were studied with scanning electron microscopy (SEM) to prove the suitability of electrospun scaffolds for cultivating neural stem cells. Subsequently, the cell viability, differentiation, proliferation, and adhesion behaviors of NSCs on 2D and 3D scaffolds were remarkably investigated. Eventually, the biocompatibility of implanted microchannels was successfully evaluated by host tissue reaction in vivo test.

## 2. Material and Methods

### 2.1. Materials and Preparation

#### 2.1.1. Materials

All reagents (sodium dodecyl sulfate (SDS), hydroxide sodium (NaOH)) and solvents (hexafluoroisopropanol (HFIP), trifluoroacetic acid (TFA), dimethyl formamide (DMF), hydrochloric acid (HCl) and ethanol (EtOH)) were purchased from Merck (Merck Millipore, Darmstadt, Germany), Sigma Aldrich (Sigma Aldrich Chemie GmbH, München, Germany) and VWR (Radnor, PA, USA) in the highest purity available and were used as received. Genipin (>99% purity) was purchased from ChemNorm (Wuhan, China). Fmoc-protected amino acids and Rink Amide resin were obtained from CEM (Matthews, NC, USA).

#### 2.1.2. Synthesis and Purification of Self-Assembly Peptides

All peptides were synthesized via microwave-assisted Fmoc SPPS on a 0.56 mmol/g Rink Amide resin using a CEM Liberty Blue system (CEM Corp., Matthews, NC, Canada) with a 0.25 mmol scale. Coupling conditions were as follows: 4 min, 90 °C, and 50 W of microwave energy. Each Fmoc-protected amino acid was dissolved in 0.2 M DMF. A solution of 1 M DIC (in DMF) as an activator and 1 M Oxyma (in DMF) solution as an activator-based were used for the coupling reaction. A solution of 10% *v*/*v* piperazine in 9:1 NMP/EtOH was used to remove the Fmoc group. The N-terminal groups were acetylated using a 20% *v*/*v* solution of Ac_2_O in DMF. The peptide derivative was finally cleaved from the resin with a mixture containing 92.5% TFA, 2.5% H_2_O, 2.5% DODt, and 2.5% TIS (*v*/*v*/*v*/*v*), precipitated in ice-cold diethyl ether. The peptide was lyophilized (Labconco, Kansas City, MO, USA) in CH_3_CN/H_2_O (25:75). The crude material was then purified via reverse phase HPLC using a Waters binary HPLC on a Restek^TM^ (Restek Corp., Bellefonte, PA, USA) Prep C18 column with a gradient of 0.1% TFA in acetonitrile and 0.1% TFA in H_2_O varying acetonitrile from 25% to 75% over 30 min. After semi-preparative HPLC purification, a solution of 0.1 M HCl was added to the isolated product in order to remove TFA residues.

The linear sequence Phe-Ala-Gln-Val-Pro-Pro-Gly-Gly-Gly-(Leu-Asp-Leu-Lys)_3_-CONH_2_ is called FAQ-(LDLK)_3_.

The mixture of linear sequences Ac-(Leu-Asp-Leu-Lys)_3_-CONH_2_, Ac-Lys-Leu-Pro-Gly-Trp-Gly-Gly-Gly-Gly-(Leu-Asp-Leu-Lys-Leu)_3_-CONH_2_, Ac-Ser-Ser-Leu-Ser-Val-Asn-Asp-Gly-Gly-Gly(Leu-Asp-Leu-Lys)_3_-CONH_2_ and branched tris(Leu-Asp-Leu-Lys)_3_-CONH_2_ is named HYDROSAP.

The self-assembling backbone of such linear SAP sequences is prone to cross-b structure formation similar to EAK16 and RADA16 [23]; however, LDLK12 has been preferred because of its strong self-assembling propensity with a shorter sequence, making it more competitive in terms of production costs, and presence of Lys residues, featuring primary amine groups which have been demonstrated to be the best reactive sites for genipin crosslinking [13].

All mass spectra were detected on an LC-MS via single quadrupole mass detection (Waters LC-MS Alliance 3100, Waters Corporation, Milford, MA, USA) using nebulizing nitrogen gas at 800 L/min and a temperature of 150 °C, cone flow 10 mL/min, capillary 3.11 kV and cone voltage 52 V.

#### 2.1.3. Crosslinked SAP Preparation

Crosslinked peptides, FAQ(LDLK)_3_gp and HYDROSAPgp powder, are prepared by adding 33 mg of Genipin dissolved in 10 mL of water:ethanol (80:20 *v*/*v*) to the 100 mg or 50 mg purified FAQ(LDLK)_3_ or HYDROSAP powders, respectively, to achieve a final 1.33% or 0.83% *w*/*v* concentration solutions. It is worth underlining that Genipin had to dissolve quietly in the ethanol initially, and distilled water was then added gently to prevent Genipin precipitation. The SAPs mixed solution was then sonicated for 30 min and incubated at 37 °C for 72 h. Finally, the dark blue SAPs crosslinked solution was flash frozen in liquid nitrogen and lyophilized at −50 °C for 72 h in a freeze dryer to achieve powdered crosslinked peptides.

#### 2.1.4. Electrospinning of Crosslinked SAPs

Electrospinning solution for SAP-based fibers was prepared by dissolving 37 wt% of FAQ(LDLK)_3_:HYDROSAP:SDS with the different ratios in a mixture of solvents containing HFIP and TFA (99:1 *v*/*v*) (Table 1). The FAQ(LDLK)_3_, HYDROSAP, and SDS were dissolved in the HFIP:TFA by being continuously vortexed to form a homogeneous solution. Subsequently, the solution was sonicated for 30 min at room temperature (bath temperature kept low by a glass of ice), and afterward, the solution was used straight away for electrospinning.

The fibers were electrospun using Electroris (FNM Ltd., Fanavaran Nano-Meghyas Company (FNM Co. Ltd.), Tehran, Iran, www.fnm.ir, accessed on 28 August 2023) as an electrospinning device having precise humidity and temperature controller. Briefly, the solutions were loaded in a syringe (diameter d = 4.6 mm, BD Micro-Fine, Becton, Dickinson and Company Corporate (BD), Franklin Lakes, NJ, USA) and placed in the horizontal direction. The positive electrode was connected to the 29 G (diameter d = 0.33 mm) syringe needle, and the negative electrode was attached to the collector. A circular flat collector covered with an aluminum sheet (diameter 7.5 cm) and rotating 33G needles mounted as a target in the rotating arm of the mandrel were used to fabricate 2D fibrous lamina and 3D fibrous microchannels, respectively. The distance between the tip and the collector was set to 80 mm, and the scanning range and rate were adjusted to 50 mm and 1000 mm/min to distribute electrospun fibers on the collectors. A controllable syringe pump in the range of 0.01–100 mL/h was used to feed the needle. The applied parameters were voltage tension = 17/18 kV, tip-collector distance = 8 cm, flow rate = 20–40 µL/h, humidity = 45% and temperature = 22 °C.

To remove trapped solvents, the electrospun mats were vacuum-dried at room temperature for one hour before post-treatment. Table 1 shows the optimal parameters applied in the production of electrospun scaffolds.

#### 2.1.5. Post-Treatment

Electrospun laminas and microchannels obtained were annealed by vapor exposure to the 1–3 mL of 25 mM solution of NaOH in H_2_O for 3 days at 37 °C. After the annealing, insoluble mats were dipped in a solution of Genepin 4% in EtOH:PBS (20:80 *v*/*v*) for one day at 37 °C. In this way, Genepin could accomplish to crosslink the self-assembled peptides, increasing their stability. Finally, the post-treated mats could be stored at 4 °C in PBS.

### 2.2. Characterization

#### 2.2.1. Scanning Electron Microscope (SEM)

SEM imaging was conducted with a Tescan VEGA TS 5136XM, TESCAN Company, Brno, Czech Republic, to investigate the samples’ morphology. All samples were sputter-coated with a nominally 20 nm thin gold film using a Quorum Tech Q150R S, Quorum Company, East Sussex, UK, sputter coater. The fiber diameters and their distribution were measured using the ImageJ 1.52a software.

#### 2.2.2. Fourier Transform Infrared (FTIR)

FTIR spectra were recorded on a PerkinElmer FTIR spectrometer in the spectral range 400–4000 cm^−1^ with a resolution of 1 cm^−1^ in transmission mode. The FTIR measurements were repeated three times at random locations for each scaffold type to minimize the possibility of error. In order to minimize the interference of water peaks and peptides in the amide I region, entire samples were dried by vacuum dryer. Data processing was performed using Origin 2020 software (OriginLab Corporation, Northampton, MA, USA). All the measured spectra are background-corrected and normalized. A peak analyzer was used to perform non-linear fitting of the peaks in the spectral data. Baseline corrections were performed using a second derivative (zeroes) method for finding anchor points and detecting the baseline. Hidden peaks were also detected in the spectral range 1500–1700 cm^−1^ by a second derivative method followed by smoothing with the ten-point Savitsky–Golay function with polynomial order of 2. The deconvoluted spectra were fitted with the Gaussian function. The positions and the number of the components (used as an input file for the curve-fitting function) were obtained from both the second derivative and the deconvoluted spectra. The quality of the fitting was estimated by standard deviation.

#### 2.2.3. Ninhydrin Test

The free amine degree, which is defined as the ratio of the free amine groups in the crosslinked samples to the free amino groups in the corresponding uncrosslinked samples, was determined by 2,2-dihydroxyindane-1,3-dione (Ninhydrin) protocol. The number of free amino groups in the test sample was determined by the optical absorbance of the solution at 570 nm recorded with a multi-modal plate reader (Tecan) using a standard curve. Lyophilized FAQ(LDLK)_3_ and crosslinked FAQ(LDLK)_3_gp samples were each dissolved at 10 mg/mL in distilled water. A linear calibration curve was created using different FAQ(LDLK)_3_ concentrations. Triplicates of unmodified FAQ(LDLK)_3_ were serially diluted in distilled water from 0 to 10 mg/mL in increments of 2 mg/mL in a 96-well plate. The crosslinked FAQ(LDLK)_3_gp sample was plated in triplicate without dilution. A 12 mM (2.2 mg/mL) solution of ninhydrin in filtered ethanol was added to each plated sample in a 1:1 *v*/*v* ratio of ninhydrin to FAQ(LDLK)_3_ solution (total volume 100 μL per well). The plate was sealed and placed in boiling water until a linear pattern of color development was observed (about 20 to 30 min). The plate was then left to cool for 10 min, and the absorbance was measured at 570 nm, which is the typical absorbance for the purple complex formed upon ninhydrin reaction with amino acids. A standard curve was generated from the mean absorbance for each FAQ(LDLK)_3_ standard. For crosslinked samples, the fraction of amines available was determined by Equation (1):

(1) $$Fraction\;of\;amines\;available=\frac{Apparent\;sample\;con.}{Nominal\;sample\;con.}$$

where the apparent concentration was obtained from the standard curve, and the nominal concentration was defined as the concentration at which the protein sample solution was prepared. The degree of crosslinking (*DoC*) was determined by Equation (2):

(2) $$DoC\;\left(\%\right)=100\times \left(1-\frac{Apparent\;sample\;con.}{Nominal\;sample\;con.}\right)$$

#### 2.2.4. Fluorescence Intensity Test

In order to determine the crosslinking rate of FAQ and HYDROSAP by Genipin, the crosslinking reactions were tracked by measuring fluorescence intensity created by blue pigmentation formation resulting from the reaction between primary amine and Genipin at different time points. An Infinite M200 PRO, Tecan Company, Männedorf, Switzerland, plate reader (Tecan) was used to record fluorescent intensity at λex = 590 nm and λem = 630 nm from 0 h to 102 h at 21 °C. All experimental runs were repeated three times for each different time point. The spectra were averaged and processed with the OriginPro software using Boltzmann fitting.

### 2.3. Neural Stem Cells (NSCs) Culture

#### 2.3.1. Two-Dimensional Scaffold In Vitro Test: Cell Viability and Differentiation Assay

Human neural stem cells (hNSCs) and murine neural stem cells (mNSCs) were established and expanded as previously described [34,38]. Briefly, human fetal brain tissue specimens derived from the forebrain were collected from spontaneous miscarriages at gestational ages greater than the 8th post-conceptional week, upon the mother giving informed, written consent, according to good manufacturing practice (GMP) protocols, in agreement with the European Medical Agency (EMA) guidelines and Agenzia Italiana del Farmaco (AIFA), protocol number aM 101/2010 (updated in 2018 after AIFA inspection to number aM 54/2018). mNSCs were isolated from the subventricular zone (SVZ) of 8-week-old CD-1 albino mice striata. SAP laminas (FAQ(LDLK)_3_gp, FAQ(LDLK)_3_gp-sds, FAQ(LDLK)_3_gp-HYDROSAPgp, and FAQ(LDLK)_3_gp-HYDROSAPgp-sds) were placed into 96-multiwell plates and cells were seeded on the top surface of each sample at the density of 3 × 10^4^ cells/cm^2^ and cultured for 7 days in vitro. Cultrex-BME substrate was used as a gold standard. hNSCs and mNSCs were differentiated in a serum-free basal medium supplemented with basic fibroblast growth factor (bFGF, 10 ng/mL). After two days, the bFGF medium was shifted to a serum-free basal medium supplemented with leukemia growth factor (LIF, 20 ng/mL, Merck) and brain-derived neurotrophic factor (BDNF, 20 ng/mL, Peprotech, Neuilly-sur-Seine, France). Fresh medium was replaced after 3 days. Cell cultures were preserved at 37 °C, 20% O_2,_ and 5% CO_2_. After 7 days of differentiation, cell proliferation was assessed via CellTiter 96^®^ Aq_ueous_ One Solution Cell Proliferation Assay (MTS assay, Promega, Madison, WI, USA): MTS solution was added to the culture media (1:5) and incubated for 1 h at 37 °C. The supernatant of each sample was quantified via Infinite M200 PRO plate reader (Tecan, Männedorf, Switzerland) by measuring absorbance at 490 nm. LIVE/DEAD™ viability/Cytotoxicity Kit (Invitrogen, ThermoFisher, Waltham, MA, USA) was used to evaluate cell viability. Live cells were shown in green with Calcein-AM, while Ethidium homodimer-1 was used to stain dead cells in red. For immunofluorescence tests, samples were fixed with 4% paraformaldehyde, washed with D-PBS, permeabilized with 0.3% Triton X-100, and blocked with 10% normal goat serum. The following primary antibodies were used: rabbit anti-GFAP (1:500, DakoCytomation, Santa Clara, CA, USA), mouse anti- bIIITubulin (1:500, Biolegend, San Diego, CA, USA), mouse anti-GalC (1:200, Merck), and mouse anti-O4 (1:200, Merck). Secondary antibodies were goat anti-mouse Alexa 488 (1:1000, Molecular Probes, Eugene, OR, USA), goat anti-rabbit Cy3 (1:1000, Jackson Immunoresearch), and goat anti-mouse Cy3 (1:1000, Jackson Immunoresearch, West Grove, PA, USA). Cell nuclei were stained with Hoechst 33342 (1:500, Molecular Probes). Three different fields from three independent experiments were randomly acquired using the Zeiss microscope ApoTome System and processed by manually counting positive cells for each marker using NIH-ImageJ 1.52a software.

#### 2.3.2. Three-Dimensional Scaffold In Vitro Test: Cell Viability and Differentiation Assay

Microchannels described above were tested in vitro. hNSCs at the density of 4.5 × 10^4^ cells/µL were encapsulated inside HYDROSAP hydrogel, previously dissolved 1% *w*/*v* in distilled water (GIBCO) and mixed with sucrose and NaOH [34,39]. The resulting mixed solution was injected inside FAQ(LDLK)_3_gp microchannels by using a Hamilton syringe. These composite scaffolds were placed in a 24-well serum-free medium filled with bFGF for the first two days. After two days, the medium was changed to basal medium supplemented with LIF and BDNF. The fresh medium was replaced every 3 days. After 2 weeks in culture, samples were fixed with 4% paraformaldehyde, embedded in OCT and cryosectioned at 50 µm. For immunofluorescence analyses, sections were permeabilized with 0.3% Triton X-100 for 10 min at 4 °C and blocked with 10% normal goat serum (GIBCO, Waltham, MA, USA) for 1 h at room temperature. In addition to the antibodies listed above, the following primary and secondary antibodies were used: mouse anti-MAP2 (1:300, Invitrogen), rabbit anti-GAP43 (1:100, Merck), mouse anti-SMI31 (1:1000, BioLegend), rabbit anti-GABA (1:500, Sigma-Aldrich) and goat anti-rabbit Alexa 488 (1:1000, Invitrogen). To reveal apoptotic cells, the TUNEL assay (in situ cell death detection kit fluorescein, Roche) was performed; sections were permeabilized with 0.3% Triton X-100 for 10 min at 4 °C and incubated with a TUNEL reaction mixture (1:10 in label solution) for 1 h at 37 °C. Fluorescence images were acquired using the Zeiss microscope ApoTome System AxioVision 4.8 and processed as previously described in the 2D cell culture section.

#### 2.3.3. Implantation of Microchannels into the Spinal Cord Tissue of Rodents (Experimental Setup)

Nine adult female Sprague-Dawley (SD) rats were employed for the study. All protocols were executed in accordance with the approval of the Institutional Animal Care and Use Committee of the University of Milan-Bicocca, and all procedures adhered to the guidelines established by the European Commission (EC) (86/609/EEC) to the Italian legislation on animal experimentation (Decreto L.vo 116/92). The animals (Harlan Laboratories, Lesmo, Italy), weighing between 250 and 275 g, were accommodated in groups of 2–3 rats per cage. They were provided unrestricted access to food and water and were maintained under a 12/12 h light/dark cycle. The implantation surgeries were conducted under meticulously sterile conditions. During the implantation procedure, rats were administered deep anesthesia through an intraperitoneal injection of ketamine (80 mg kg^−1^) and xylazine (10 mg kg^−1^). Once unresponsive to a toe pinch, the dorsal area was shaved, and a dorsal laminectomy was carried out, revealing the dura that covers the spinal cord at the thoracic level T9–T10. The vertebral column was stabilized by clamping the column at vertebra T8 and T11. Longitudinal cuts were made in both the dura and the underlying spinal cord (sham group). Three microchannels (each measuring 3 mm in length), all of the same type, were introduced into the incision site located at the thoracic level T9–T10. We tested the following microchannels: FAQ(LDLK)_3_gp and FAQ(LDLK)_3_gp electrospun. After the implantation procedure, the muscle overlying the area and the skin were sutured using vicryl sutures and metal clips, respectively. The animals were observed for a duration of six weeks post-implantation, during which no notable behavioral alterations or adverse effects were noted. Rats were treated daily for one week with an analgesic (carprofen, 5 mg kg^−1^) and an antibiotic (enrofloxacin, 5 mg kg^−1^).

#### 2.3.4. Immunohistochemistry

Six weeks after implantation, rats were subjected to deep anesthesia using an overdose of avertin (400 mg kg^−1^). The animals were euthanized through cardiac perfusion under terminal anesthesia utilizing PFA 4%. After removal, the spinal cords were subjected to overnight post-fixation in 4% PFA. Subsequently, the tissues were preserved in a 30% sucrose solution and then sliced into 20 μm thick longitudinal sections using a cryostat with a frozen blade. These sections were serially cut, with three sections placed on each glass slide. To stain for macrophages and microglia, the sections were first washed with PBS, then permeabilized using 0.1% Triton X-100, and subsequently treated with 10% normal goat serum. The primary antibodies employed were mouse anti-CD68 (diluted at 1:500, Serotec, Hercules, CA, USA) and rabbit anti-IBA1 (diluted at 1:1000, Wako, Richmond, VA, USA). To conduct an immunofluorescence analysis of gliosis, a mouse anti-glial fibrillary acidic protein (GFAP) antibody was employed (diluted at 1:500, Millipore, Darmstadt, Germany). To visualize the primary antibodies, secondary antibodies were utilized as follows: goat anti-rabbit Cy3 (diluted at 1:1000, Jackson) and goat anti-mouse Alexa 488 (diluted at 1:1000, Invitrogen). The sections were counterstained using DAPI and were then mounted using FluorSave (Calbiochem, San Diego, CA, USA). To ensure consistency in the results, all measurements were conducted using digital images captured with a Zeiss Apotome microscope at a magnification of 20×. The measurements were carried out using ImageJ 1.52a software. The assessment of the reactivity area for GFAP, IBA1, and CD68 at the implantation site was conducted on longitudinal sections utilizing ImageJ software. Color images of cells expressing these markers were transformed into binary images, and the areas were quantified by counting the number of positive pixels. The pixel area was transformed into a percentage of the reactivity area, and these measurements were averaged across all sections for each animal. This approach was employed to quantify the reactivity area of each scaffold for every individual animal.

### 2.4. Statistical Analysis

Data were processed using Excel, GraphPad Prism 8, and OriginPro 1.52a software. Reported values are as means ±standard error of the mean (SEM). All experiments were repeated three times. Secondary structures were analyzed using One-way ANOVA (paired comparison plot), and Tukey’s post hoc test was used for comparative analysis and statistical significance, delineated as * *p* ≤ 0.05, ** *p* ≤ 0.005, and *** *p* ≤ 0.0005.

For in vitro studies, the MTS assay was processed through one-way ANOVA followed by Dunnett’s multiple comparison tests; βIII-Tubulin and GFAP were evaluated by two-way ANOVA followed by the Bonferroni’s multiple comparison test, and GalC-O4 was performed via one-way ANOVA followed by Dunnett post-test. In the in vivo study, IBA1/CD68 significance tests were carried out by two-way ANOVA followed by the Bonferroni post-test; finally, the GFAP marker was analyzed by one-way ANOVA followed by Tukey’s multiple comparison test.

## 3. Result

### 3.1. Morphology

**Figure 1:**
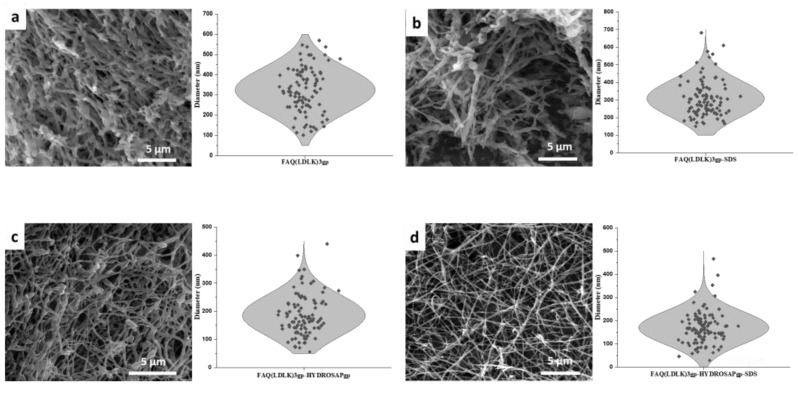
SEM images of electrospun 2D scaffolds w/o post-treatment include fibers diameter distribution: (**a**) electrospun FAQ(LDLK)3gp with diameter distribution between 100 to 600 nm and an average diameter of 324.8 ± 11.1, (**b**) electrospun FAQ(LDLK)3gp-sds shows narrower diameter distribution with lower average diameter 307.9 ± 10.3 nm, (**c**) electrospun FAQ(LDLK)3gp-HYDROSAPgp shows diameter distribution between 80–400 nm with an average diameter of 185.3 ± 7.3 nm, and eventually (**d**) FAQ(LDLK)3gp-HYDROSAPgp-sds, comprising surfactant and mixed peptides, shows very narrow diameter distribution between 50–300 nm with an average diameter of 169.8 ± 7.0 nm.

Figure 1 demonstrates the images and morphological profiles of four electrospun 2D scaffolds consisting of different components without post-treatment. Initially, we tested different electrospinning solutions (solvents, concentration, and solvent/cosolvent ratio) and, thus, the fiber diameter; by adjusting the electrospinning conditions, especially the polymer concentration and the solvents used, we obtained roughly round FAQ(LDLK)_3_gp nanofibers that displayed minimal beading and a highly fibrous structure.

SDS, an anionic surfactant, was added to the polymer solution to investigate its effects on fiber diameter and morphology. These FAQ(LDLK)_3_gp-sds fibers spun from solution with SDS were more uniform in diameter and had fewer beads compared to FAQ(LDLK)_3_gp fibers prepared w/o surfactant additive. The SEM images and morphological profile of nanofibers electrospun from the FAQ(LDLK)_3_gp solution with and w/o SDS are shown in Figure 1a,b, where both the bead content and the average diameter decreased slightly from 324.8 ± 11.1 to 307.9 ± 10.3 nm after the addition of SDS.

On the other hand, the impact of HYDROSAPgp as multi-functionalized SAPs with specific characteristics on the morphology of electrospun FAQ(LDLK)_3_gp nanofibers was assessed. It is shown that the addition of 10% HYDROSAP not only improves the spinnability of the SAP solution but can also reduce fiber sizes and bead content significantly. Figure 1c indicates a narrow diameter distribution for electrospun FAQ(LDLK)_3_gp-HYDROSAPgp nanofibers with average diameters of around 185.3 ± 7.3 nm.

**Figure 2:**
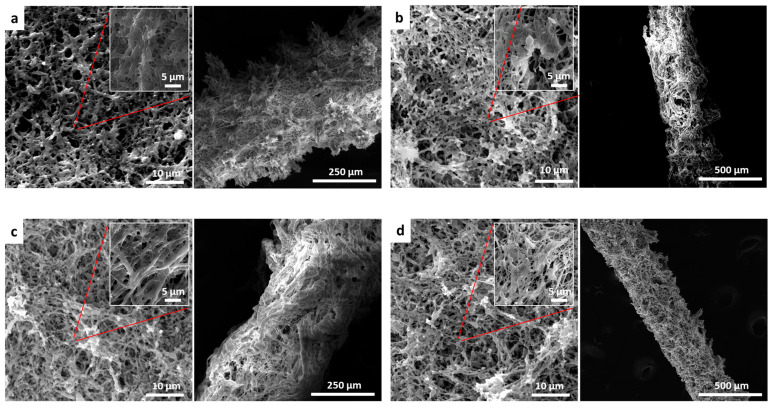
SEM images of electrospun 2D and 3D scaffolds after post-treatment (annealing and crosslinking): (**a**) electrospun FAQ(LDLK)3gp lamina and channel show rough surface with fibers connection, (**b**) electrospun FAQ(LDLK)3gp-sds lamina and channel with more uniformity of fibers and surface after adding a surfactant, (**c**) electrospun FAQ(LDLK)3gp-HYDROSAPgp lamina and channel comprise defect-free fibers and even surface, (**d**) electrospun FAQ(LDLK)3gp-HYDROSAPgp-sds lamina and channel reveals very uniform fibers and surface after adding SDS and HYDROSAP (left: 2D lamina, right: 3D microchannels).

Modification strategy based on Genipin crosslinking that was applied on scaffolds to increase their mechanical properties influenced scaffold surface morphology. SEM images reveal significant changes in scaffold surface topography due to crosslinking reaction (Figure 2). As a matter of fact, the post-treatment reaction has enhanced fibers’ connection through inter-polymer chain connections or crosslinking.

SEM images of 3D microchannels demonstrate porous construction and the structure of the electrospun fibers around the microchannels. More uniform microchannels can be seen when SDS and HYDROSAP are added to the FAQ(LDLK)_3_gp solution. This nanofibers scaffold creates an open network of paths for nutrient transport into and out of the channel.

### 3.2. Genipin Crosslinking

The reaction between peptides and Genepin is well understood for a variety of experimental conditions and yields composites and complexes with no cytotoxicity for human and animal cells [40].

**Figure 3:**
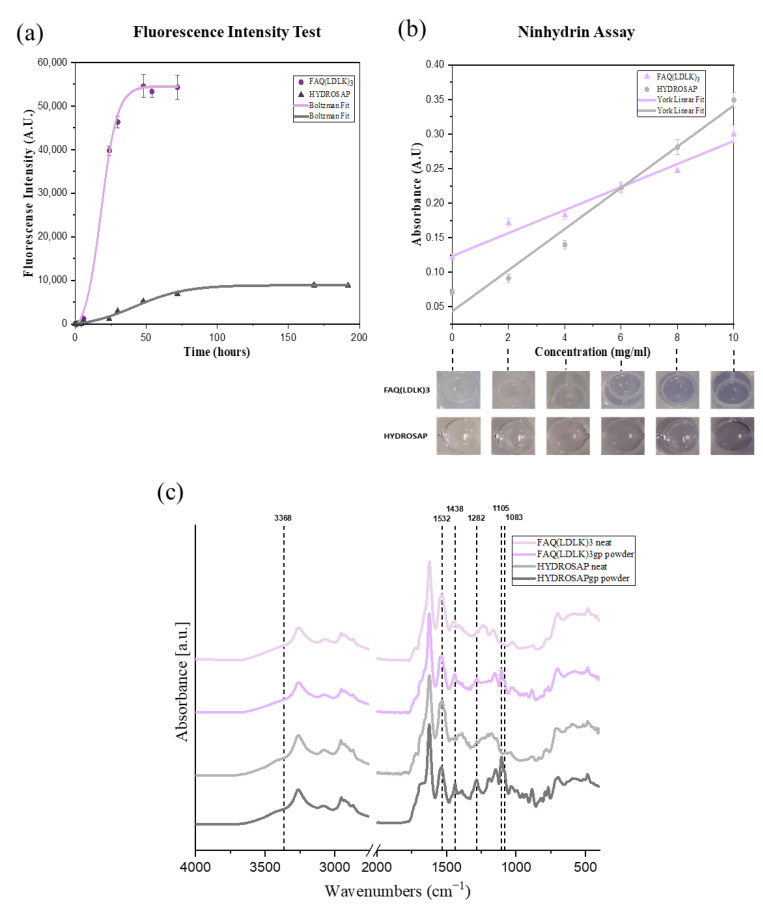
The crosslinking reaction progress was assessed using (**a**) Fluorescence intensity test by tracking the fluorescence intensity of blue fluorescent pigmentation generated by Genipin reaction with primary amines in peptides at 630 nm at different time points, (**b**) Ninhydrin assay by measuring the loss of free amine groups, reacted with ninhydrin, based on optical absorbance measurements at 570 nm, and (**c**) FTIR analysis to delineate the chemical and secondary structure changes in peptides after crosslinking by Genipin. The spectra were averaged and processed with the OriginPro software using Boltzmann fitting and York linear fitting.

Crosslinking reaction progress was assessed by tracking the fluorescence intensity of blue fluorescent pigmentation generated by the Genipin reaction with primary amines in peptides and proteins as a bi-functional crosslinking compound that correlates with the degree of crosslinking [41]. Figure 3a shows fluorescence intensity at 630 nm at different time points. In the early hours of the reaction, the crosslinking solution turned gradually from colorless to light blue, leading to a concomitant increase in fluorescence intensity. As the crosslinking progresses, the color of the crosslinking solution changes to a darker blue, resulting in more intensive fluorescence. After 72 h, fluorescence intensity reached the highest value and remained constant because a stable blue pigmentation had been formed in FAQ(LDLK)_3_gp hydrogels.

Reactive groups involved in crosslinking are the amino groups on lysine. Thus, the extent of crosslinking can be represented by measuring the loss of free amine groups using a Ninhydrin assay based on optical absorbance measurements at 570 nm. The Ninhydrin test is a chemical test performed to detect the presence of ammonia, primary/secondary amines, or amino acids. This test involves the addition of a ninhydrin reagent to the test sample that results in the formation of a purple complex in the presence of an amino group [42,43].

We applied the optimized reaction conditions and tested the dynamic range of the assay in a 96-well plate. As expected, no precipitation was observed, and the reaction became visibly purple at higher FAQ(LDLK)_3_ and HYDROSAP concentrations. A linear absorbance regression was obtained from 0 to 10 mg/mL FAQ(LDLK)_3_ solutions (R^2^_FAQ(LDLK)3_ = 0.9881 and R^2^_HYDROSAP_ = 0.9879) (Figure 3b).

The intensity of the purple color in the crosslinked sample, FAQ(LDLK)_3_gp, was lower because fewer free amines were present. To determine the fraction of amines remaining in a sample of FAQ(LDLK)_3_gp, the “apparent” solution concentration was determined from the standard curve and normalized to the nominal concentration of the sample (Equation (1), Section 2). DoC was defined as the difference of this value from unity (Equation (2)). For example, a FAQ(LDLK)_3_gp sample prepared at 10 mg/mL was reacted with ninhydrin and yielded an apparent concentration of 0.14 mg/mL for a calculated DoC of 98.6%.

In addition, FT-IR was used to delineate the chemical changes in peptides after crosslinking by Genipin, as shown in Figure 3c. The reaction between primary amine groups of FAQ(LDLK)_3_ and HYDROSAP with Genipin has been investigated in the different boundary regions of the IR spectrum. The spectrum of FAQ(LDLK)_3_ and HYDROSAP revealed that the peak at 3368 cm^−1^ appeared as an amine group stretching vibration mode of peptide bonds (Amide A (3300–3400)). The absorption band at 1532 cm^−1^ was the characteristic peak for amide II (N-H bending vibration). The peaks of 1083 and 1105 cm^−1^ were characteristic of the C-N stretching vibration of aliphatic amine. Compared with the non-crosslinked peptides’ spectrum, the absorption peaks of Genipin-crosslinked peptides decreased at 3368 and 1532 cm^−1^ and increased at 1083 and 1105 cm^−1^ due to Genepin crosslinking. This incident is interpreted as the formation of C–N bonds at the expense of C–O bonds during the formation of the heterocyclic Genipin–peptide compound that is evident by a new peak around 1438 cm^−1^ attributed to ring-stretching of heterocyclic amine after crosslinking by Genipin. It is interpreted that the olefinic carbon atom at C-3 of Genipin under nucleophilic attack by the amino group of peptides leads to the opening of the dihydropyran ring and formed a tertiary amine [40,44]. Meanwhile, the absorption peak at 1282 cm^−1^ corresponded to C-N stretching coupled with N-H in-plane bending vibrations increased (Amide III (1200–1350)- α-helix) after crosslinking by Genipin, ChemNorm (Wuhan, China).

### 3.3. Secondary Structure Components (FTIR)

**Figure 4:**
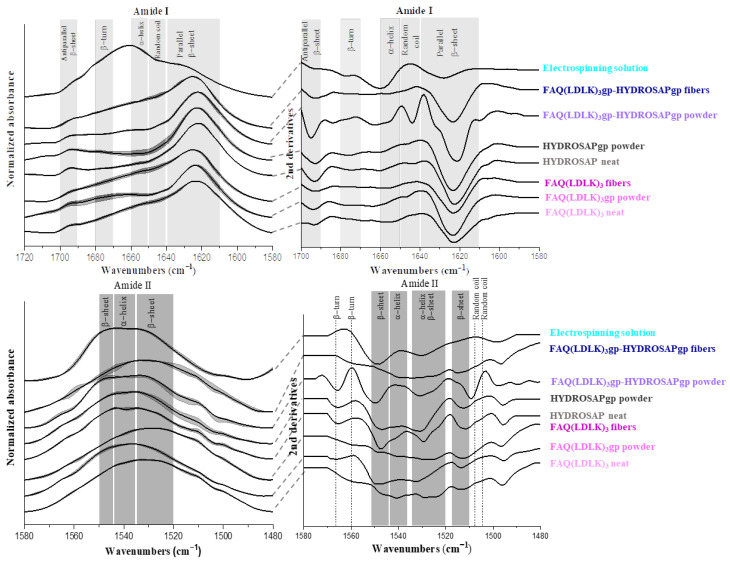
FTIR spectra along with 2nd derivatives in amide I and II absorption region of entire samples: amide I (**Up**), Amide II (**Down**). Concerning amide I mode, the entire spectra, except the electrospinning solution, demonstrate the prominent absorption peaks in the region of β-sheet centered ~1630 cm^−1^. Electrospinning solution spectra show the main peak located in the region of α-helix. the spectral region corresponding to the amide II shows a broad peak in the region 1520–1545 cm^−1^ assigned to β-sheet and α-helix. Wavenumber ranges of the principal bands characteristic of peptide secondary structure [45,46].

FTIR was used to analyze the secondary conformation of the samples. The amide I and II bands in the infrared spectrum of a peptide are the most useful probes of its secondary structure determination in solids and solutions. The specific spectral region corresponding to the amide I band is between 1600 and 1700 cm^−1^ and is mainly the result of the carbonyl (C = O) stretching mode of the peptide linkages. The bands observed in regions 1650–1660 cm^−1^ and 1640–1650 cm^−1^ generally signify α-helix and unordered structures, respectively [45,46]. The bands in regions 1620–1640 cm^−1^ and 1690–1700 cm^−1^ are assigned to a β- sheet [47]. The amide I band in regions 1670–1690 cm^−1^ is assigned to the β-turn [47]. The in-plane N-H bending vibration couples with the C-N stretching mode so that the amide II band appears in region 1480–1575 cm^−1^. The absorption peaks in regions 1520–1535 cm^−1^ and 1545–1550 cm^−1^ are attributed to the β-sheet, and regions 1535–1545 cm^−1^ are concerning α-helix secondary structure [45,46,47]. The amide I and II bands are sensitive to conformational changes from a self-assembling (non-covalent) or crosslinking bond (covalent). In the following, secondary structures of FAQ(LDLK)_3_ and HYDROSAP will be tracked throughout the process from synthesis to nanofibers fabrication. Crosslinked and non-crosslinked FAQ(LDLK)_3_ and HYDROSAP, in states of powder, solution, and fibers, with and w/o mixing, are discussed with the assignment of the band positions to an α-helix, β-sheet, β-turns, and unordered structures in their FTIR spectra. Figure 4 shows IR spectrums of entire samples in amide I and II regions. Deconvolution and second-derivative procedures were used to facilitate finding the peak positions of the amide I band in their IR spectrums and quantitative analysis of the secondary structure components (Figure S1).

Figure 4 showed a broad secondary structure in the region of amide I that indicates the presence of α-helix, β-sheet, beta-turns, and random coil, but a broad band was centered at about 1621–1626 cm^−1^ in the whole samples due to the dominant β-sheet conformation as the main amide I component that was confirmed by deconvoluted peaks as well (Supporting Information Figure S1). Another amide I bands appeared in the 1652–1658 cm^−1^, 1671–1679 cm^−1^, 1643–1647 cm^−1^, and 1692–1695 cm^−1^ ranges due to α-helix, β-turns, unordered structures, and anti-parallel β-sheet respectively. After profound investigation, except for the disappeared peak at 1645 cm^−1^ concerning random coil, for electrospun peptides, there was no significant peak shifting given by scaffold extra processing, namely crosslinking, electrospinning, and mixing peptides (peak shifting range 0–6 cm^−1^). On the other hand, amide II bands appeared in 1510–1550 cm^−1^ for the whole samples, attributable to the α-helix and β-sheet. In sample FAQ(LDLK)_3_, the amide II mode appeared near 1525 cm^−1^ at the wavenumber value typical of β-sheet, but in crosslinked peptides (FAQ(LDLK)_3_gp), this band shifted to higher wavenumbers toward α-helix region about 1535 cm^−1^. In electrospun peptides (FAQ(LDLK)_3_gp fibers), a stronger peak appeared at 1542 cm^−1^ in comparison to FAQ(LDLK)_3_gp. With regards to HYDROSAP peptides, a weaker peak existed in the range of α-helix after crosslinking with Genipin.

**Figure 5:**
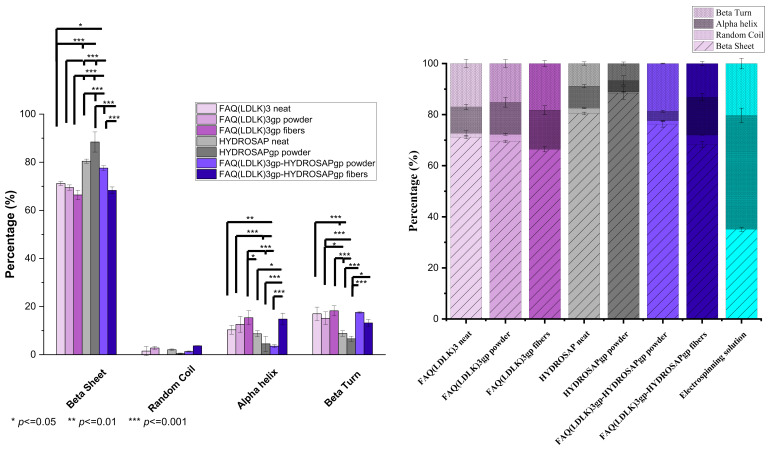
Secondary structure content measured by three mathematical resolution enhancement methods of Fourier self-deconvolution, second derivative analysis, and band curve-fitting. Data are represented as average ±SEM (N = 3). Statistical analysis: One-way ANOVA followed by Tukey multiple comparison test. Statistical analysis shows significant differences between conditions (* *p* ≤ 0.05, ** *p* ≤ 0.01, and *** *p* ≤ 0.001) (**Left**), and the bar chart represents percentages of secondary structures as determined by the method of curve-fitting and peak deconvolution (**Right**).

The qualitative results on secondary structure content were measured by curve-fitting and peak deconvolution. Figure 5 displays the percentages of secondary structure content. The data confirmed that FAQ(LDLK)_3_ had a prevailing β-sheet structure (~71.15%), with about 16.99% of β-turn, 10.33% of α-helix, and 1.5% unordered structure content. Among these samples, HYDROSAPgp showed the highest β-sheet content (~88.42%) that could improve β-sheet content in FAQ(LDLK)_3_gp-HYDROSAPgp powder and electrospun fibers to 71.74% and 68.3%, respectively, compared to the counterpart without HYDROSAP (*** *p* ≤ 0.0005). Genipin crosslinking revealed no significant differences in secondary structures content of FAQ(LDLK)_3_gp in comparison with un-crosslinked FAQ(LDLK)_3_. In contrast, crosslinking treatment of HYDROSAP showed a significant increase in β-sheet formation by about 8% (*** *p* ≤ 0.0005). As expected, electrospun FAQ(LDLK)_3_gp-HYDROSAPgp revealed significant differences in secondary structure content, ~11% decrease for β-sheet (*** *p* ≤ 0.0005), and ~14% and ~4% increase for α-helix and β-turn (*** *p* ≤ 0.0005), respectively. It can be likely due to changes in molecular order in fibers caused by electrostatic force. Nevertheless, the prevailing component of the electrospun SAPs is still β-sheet, in agreement with previous data we reported [27]. On the contrary, electrospun FAQ(LDLK)_3_gp did not indicate significant differences in comparison with FAQ(LDLK)_3_gp powder. Moreover, adding 10% *w*/*w* HYDROSAPgp to FAQ(LDLK)_3_gp showed a significant increase in β-sheet and reduction of α-helix (*** *p* ≤ 0.0005). Concerning the electrospinning solution, it revealed a significant increase in α-helix content, which composes the major part with ~50%.

### 3.4. Two-Dimensional and Three-Dimensional In Vitro Cell Cultures

**Figure 6:**
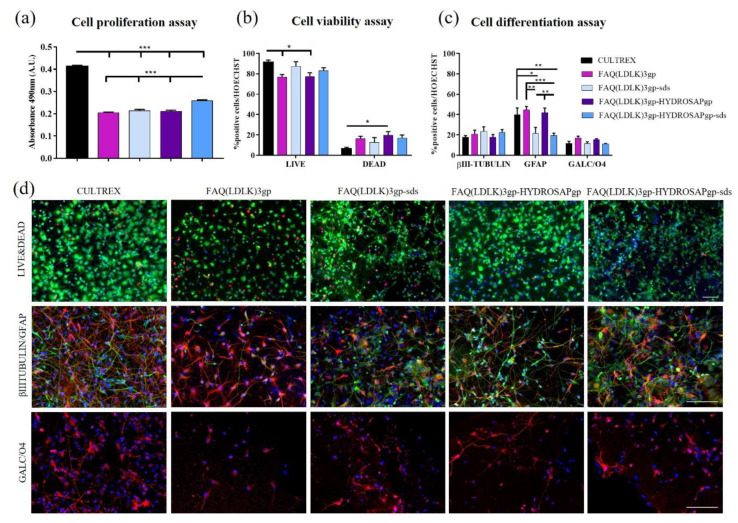
Proliferation, viability, and differentiation assays of hNSCs seeded on 2D scaffolds of FAQ(LDLK)3gp, FAQ(LDLK)3gp-sds, FAQ(LDLK)3gp-HYDROSAPgp and FAQ(LDLK)3gp-HYDROSAPgp-sds after 7 days in vitro. (**a**) Colorimetric MTS assay for cell proliferation assessment. (**b**) LIVE/DEAD Cell Viability/Cytotoxicity test to determine cell viability. (**c**) Immunostainings for βIII-Tubulin (neurons in green), GFAP (astrocytes in red), and GALC/O4 (oligodendrocytes in red) markers. (**d**) Representative fluorescence images for cell viability assay (top), neural and astroglial differentiation (middle), and oligodendroglial differentiation (bottom). Live cells are labeled in green, and dead cells in red. Cell nuclei were stained with HOECHST (in blue). Data are represented as mean ± SEM. Statistical analysis shows significant differences between conditions (* *p* < 0.05; ** *p* ≤ 0.01; *** *p* < 0.001). All measures were performed in triplicate. Scale bar, 100 µm.

In vitro investigations were conducted using the manufactured electrospun FAQ(LDLK)3gp-based nanofibrous mats to examine cell behavior in terms of proliferation, viability, and differentiation. We studied the proliferation, viability, and morphology of differentiated hNSCs (after 7 days in vitro) seeded on four types of electrospun nanofibrous mats: FAQ(LDLK)_3_gp, FAQ(LDLK)_3_gp-sds, FAQ(LDLK)_3_-HYDROSAPgp and FAQ(LDLK)_3_-HYDROSAPgp-sds. Cultrex was used as a positive control. Cell proliferation and viability were assessed with an MTS assay (Figure 6a). Cells seeded on gold standard Cultrex produced the highest value (0.41 ± 0.01 Absorbance A.U) when compared to treated samples (*** *p* < 0.001). Nevertheless, FAQ(LDLK)_3_-HYDROSAPgp-sds (0.26 ± 0.01 A.U.) significantly exceed (*** *p* < 0.001) all other samples: 0.20 ± 0.003 A.U. for FAQ(LDLK)_3_gp, 0.21 ± 0.01 A.U. for FAQ(LDLK)_3_gp-sds, and 0.21 ± 0.005 A.U. for FAQ(LDLK)_3_-HYDROSAPgp. A higher proliferation rate in the Cultrex sample is due to its high content of extracellular matrix proteins, such as laminin, collagens, entactin, and heparan sulfate proteoglycans, plus growth factor content incorporated in the culture medium. All these combinations provide structural support for cells and play an important role in establishing tissue organization by influencing cell adhesion, proliferation, and differentiation. LIVE/DEAD fluorescence assay was used to confirm cell viability (Figure 6b). The percentage of live cells slightly decreased (* *p* < 0.05) on FAQ(LDLK)_3_gp (77.06 ± 2.35%) and FAQ(LDLK)_3_-HYDROSAPgp (77.28 ± 3.82%) compared to Cultrex (91.14 ± 2.47%); on the contrary, SDS treatment seems to not affect the viability of differentiated hNSCs with values between 87.3 ± 4.67 for FAQ(LDLK)_3_gp-sds, and 83.02 ± 2.98% for FAQ(LDLK)_3_-HYDROSAPgp-sds. hNSC progeny differentiation at 7 days in vitro was tested by staining neurons with βIII-Tubulin marker, astrocytes with GFAP, and oligodendrocytes with GALC/O4 (Figure 6c,d). The percentage of neurons was comparable to Cultrex, with no significant differences across groups, obtaining 17.77 ± 1.67% for Cultrex and 23.55 ± 4.46% for FAQ(LDLK)_3_gp-sds. On the contrary, proportions of astrocytes were 39.99 ± 6.63% for Cultrex, 44.44 ± 3.4% for FAQ(LDLK)_3_gp, 21.38 ± 09% for FAQ(LDLK)_3_gp-sds, 41.95 ± 4.54% for FAQ(LDLK)_3_-HYDROSAPgp and 19.53 ± 2.24% for FAQ(LDLK)_3_-HYDROSAPgp-sds. SDS addition affected astrocyte progeny by decreasing significantly GFAP + cells, compared to the counterpart without SDS (** *p* < 0.01). Nonetheless, there was no statistical evidence of significant differences between Cultrex, FAQ(LDLK)_3_gp, and FAQ(LDLK)_3_-HYDROSAPgp. The oligodendrocytes population showed statistically similar percentages in all groups: 11.73 ± 2.05% for Cultrex, 17.15 ± 1.68% for FAQ(LDLK)_3_gp, 11.48 ± 1.83% for FAQ(LDLK)_3_gp-sds, 15.25 ± 1.04% for FAQ(LDLK)_3_-HYDROSAPgp, and 11.14 ± 0.45% for FAQ(LDLK)_3_-HYDROSAPgp-sds. Similar results obtained for hNSCs were achieved for mNSCs seeded on electrospun FAQ(LDLK)_3_gp-based nanofibrous mats (Figure S2). Indeed, as depicted in Figure 6d and Figure S2, all electrospun FAQ(LDLK)_3_gp-based nanofibrous mats fostered anchoring, sprouting, and branching of differentiated hNSCs and mNSCs.

**Figure 7:**
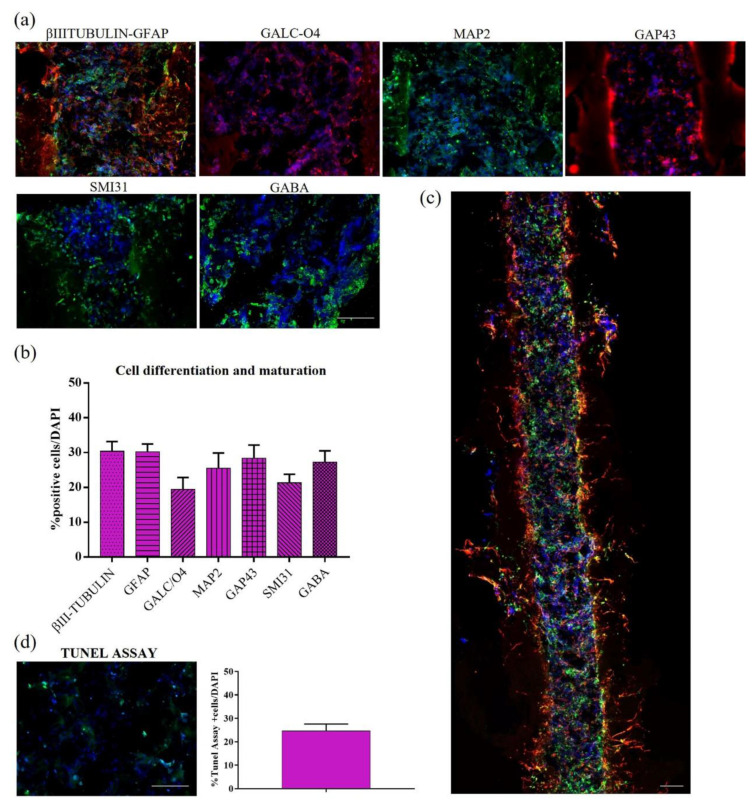
Cell differentiation and maturation of hNSCs seeded in HYDROSAP hydrogel and injected into the FAQ(LDLK)3gp microchannels (2 weeks in vitro). (**a**) Longitudinal sections of microchannels seeded with hNSCs. Neurons are labeled in green with βIIITubulin marker and astrocytes in red with GFAP; oligodendrocytes are stained with GALC/O4 in red, mature neurons in green with MAP2, growth-cones associated protein GAP43 in red, phosphorylated neurofilaments with SMI31 in green and GABAergic neurons in green. (**b**) Quantitative evaluation of neural markers for cell differentiation and maturation: βIII-Tubulin, GFAP, GALC-O4, MAP2, GAP43, SMI31, and GABA. (**c**) Full longitudinal section of a microchannel seeded with differentiated hNSCs progeny (βIII-Tubulin and GFAP stainings). (**d**) Tunel assay for apoptotic cells (green) inside the microchannels and their quantification. Cell nuclei are stained in blue with HOECHST. Data are represented as mean ± SEM (*n* = 8). Scale bars, 100 µm.

Here, we investigated the differentiation and maturation of injected hNSCs inside these 3D composite bioprostheses (see Section 2 for details). Inside the microchannels, hNSC progeny showed spread and branched morphologies (Figure 7a), with precise proportions of differentiated neural cells at different stages of maturation (Figure 7b). After 14 days in culture, we found 30.41 ± 2.76% of βIII-tubulin+ neurons, 30.21 ± 2.26% of astrocytes stained with GFAP marker, 19.43 ± 3.42% of GALC/O4+ oligodendrocytes cells, 25.51 ± 4.39% of mature MAP2+ neurons, 28.37% of elongating axons marked with GAP43, and 27.23 ± 3.29% of GABAergic neurons. The whole longitudinal section (Figure 7c showed an excellent distribution of differentiated hNSCs (stained with βIII-tubulin and GFAP markers), obtaining a 3D construct resembling a nerve conduit. To conclude the in vitro characterization, cellular apoptosis was assessed through Tunel Assay (Figure 7d). The percentage of apoptotic cells was 24.63 ± 2.95%, similar to that previously reported in 3D hNSC cultures in plain HYDROSAP gels [34].

### 3.5. Biocompatibility (Animal Testing Outcomes)

**Figure 8:**
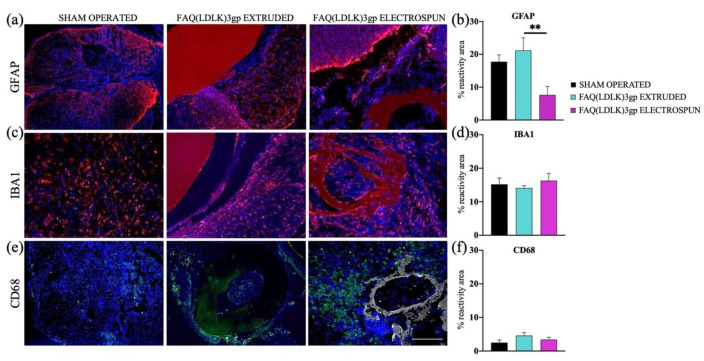
In vivo tests conducted to assess tissue response in sham-operated animals and those receiving FAQ(LDLK)3gp extruded and FAQ(LDLK)3gp electrospun scaffolds. Immunofluorescence staining: (**a**,**b**) GFAP, (**c**,**d**) IBA1, and (**e**,**f**) CD68 markers. Cell nuclei are made visible using DAPI staining (Blue). The reactive areas of GFAP+ cells (shown in red) detected near the implantation sites showed a significant difference between the FAQ(LDLK)3gp extruded and FAQ(LDLK)3gp electrospun experimental groups (** *p* ≤ 0.01). In the case of IBA1 and CD68 markers, the results on their reactivity areas showed no statistical differences among the experimental groups. Scale bar, 100 μm.

To evaluate the response of the host tissue to the introduction of empty electrospun microchannels, they were intramedullary implanted in rats and analyzed after a duration of six weeks (refer to the Method section for more information). Data were also compared to extruded (smooth) FAQ(LDLK)_3_gp channels to determine the impact of scaffold micro-topography vs tissue reaction. We evaluated the implants and the surrounding spinal cord tissues. Histological assessment was employed to investigate the impact of scaffolds on the activation of inflammatory cells. We examined the percentage of reactive area indicated by GFAP, IBA1, and CD68 markers in the spinal cord tissue adjacent to the implants. At the 6-week stage, GFAP staining revealed the presence of reactive astrocytosis encircling each implant. (Figure 8). GFAP immunoreactivity was 17.7 ± 2.1%, 21.2 ± 3.8%, and 7.6 ± 2.6% in sham-operated, FAQ(LDLK)_3_gp extruded and FAQ(LDLK)_3_gp electrospun groups respectively.

The gliosis response for FAQ(LDLK)_3_gp extruded and FAQ(LDLK)_3_gp electrospun scaffolds were significantly different. After 6 weeks, the extruded scaffold had higher gliosis than the electrospun group (** *p* < 0.01, FAQ(LDLK)_3_gp extruded vs FAQ(LDLK)_3_gp electrospun).

IBA1 functions as a calcium-binding protein specific to microglia and macrophages: the reactivity areas of IBA1 in the FAQ(LDLK)_3_gp extruded and FAQ(LDLK)_3_gp electrospun groups do not exhibit significant variations with respect to the sham group. Similarly, immunostainings for CD68+ cells revealed no noteworthy distinctions across all experimental groups.

## 4. Discussion

Although stem cell therapy has emerged as one of the most promising spinal cord injury treatments for axonal regeneration, and the therapeutic efficacy of hNSCs has previously been established in clinical transplantation protocols [48], the design of a suitable scaffold mimicking ECM chemically, physically, and mechanically remains a challenge.

On the other hand, it is observed that three-dimensional cell cultures are leading the way to the fabrication of tissue-like constructs useful to developmental biology and pharmaceutical screenings. SAP electrospun scaffolds have been frequently used for regenerative medicines due to their morphological similarities with the ECM and tunable chemical and physical properties for regulating cell behaviors and functions.

This study characterizes the influence of surfactant and multi-functionalized SAPs, which impacts the formation and morphology of the nanofibers formed during the electrospinning process. The addition of both SDS and HYDROSAP led to the formation of tiny, fibrous, and uniform nanofibers without beads-on-string morphology and with a very narrow diameter distribution between 100–300 nm with an average diameter of 169.8 ± 7.0 (Figure 1d).

Indeed, as widely reported, an anionic surfactant is capable of changing the conductivity and net charge density of the polymer solution, parameters that can strongly influence the final morphology of the fibers [49,50]. The electrical field force applied on the charged jet increased with the increase in the net charge density, which also enhanced the whipping instability and the spiral motion of charged jets. The whipping motion of the charged jet promotes the stretching process, decreasing the diameter of the fiber and preventing bead formation [49].

It is worth explaining that in the case of the system containing HYDROSAP, due to branched peptides, a new arrangement of chains occurs, which can cause additional entanglement of polymer macromolecular chains in the solution. Thus, sufficient chain entanglement allows for the formation of more uniform and defect-free fibers [51].

The IR spectra of entire samples (instead of electrospinning solution) indicated mostly similar peaks, showing that extra processing did not alter peptide secondary structures significantly. Lastly, deconvoluted spectra and second derivatives displayed the main component assigned to the β-sheet conformation, with the presence of lower amounts of α-helix, β-turn, and disordered structures. The main contribution to the secondary structure of crosslinked peptides is distributed between β-sheets and β-turns. In contrast, the electrospun samples led to higher contributions from the β-sheet and α-helix, which can reflect that forced assembly and standard self-assembly have been the driving forces responsible for secondary structure changing [27,30]. The outcomes indicated that the electrostatic force itself significantly alters the secondary structures of the peptides, but the subsequent crosslinking does not significantly influence them. The influence of the HFIP/TFA solvents on the peptide structure in the electrospinning solution was monitored using FTIR spectra.

The solvents used may promote the formation of secondary structures observed in the electrospun peptides, such as β-sheets in proteins/peptides with a primary sequence that favors this conformational arrangement.

However, most of the existing electrospun nanofibers have a closely packed two-dimensional (2D) membrane with the intrinsic shortcomings of limited cellular infiltration, restricted nutrition diffusion, and unsatisfied thickness [20,52].

3D electrospun nanofiber-based scaffolds could provide stem cells with 3D microenvironments and biomimetic fibrous structures for tissue engineering applications, as well as unique alternative ways for neural regeneration [52].

NSCs were chosen for in vitro testing because they have been well-characterized on SAP scaffolds [33,34,39,53], which were employed to investigate the Genipin-crosslinked scaffolds [13,54].

The in vitro results in 2D conditions demonstrated negligible cytotoxicity, adequate cell differentiation into the three main neural phenotypes, as well as a remarkable cell sprouting, encouraging us to extend the in vitro studies in a 3D setup and to test electrospun FAQ(LDLK)_3_gp-based nanofibrous scaffolds for in vivo biocompatibility tests.

To rebuild the neural circuitry, transplanted cells and/or regenerating nervous fibers require pro-regenerative substrates [55,56]. *As* previously established, hNSCs embedded into HYDROSAP can differentiate and maturate, showing an entangled neural network with electrically active neurons [34]. Responses from activated microglia and macrophages were employed to examine the inflammatory reaction to the implanted biomaterials. Macrophages represent the predominant cells in chronic inflammation in response to implanted biomaterials [57,58,59]. Following six weeks, IBA1–CD68 positive cells were identified in all groups, including the sham-operated tissue, primarily ascribed to the incision procedure carried out on the spinal cord during the implantation process. According to the literature. The response of astrocytes after surgery is distinguished by an increase in the number of cell bodies and their enlargement, indicative of hyperplasia [60,61,62,63]. GFAP + Cells were observed along the perimeters of the scaffold regions, creating a distinct glial boundary. This response frequently takes place subsequent to the microglial response and is linked with a cytotropic process [64,65]. Noteworthy distinctions were evident among the extruded and electrospun groups, with the extruded scaffold accounting for the majority of the GFAP reactivity rather than the implanted electrospun biomaterials.

Taking advantage of the biomimetic properties of SAPs, electrospun nanofibers were successfully functionalized to reduce glial cells and enhance neuronal differentiation in vitro [66,67,68,69].

These data suggest that the implanted scaffolds may not exacerbate the chronic inflammatory response to that one caused by the standard injury required for their implantation, whereas electrospun microchannels may provide less astrocytosis and likely better in vivo engraftment, making them promising candidates for tissue engineering therapies.

## 5. Conclusions

Electrospun self-assembling peptide-based 2D/3D scaffolds are promising ECM substitutes for damaged tissues. Further, being fully synthetic and making use of standard solid-phase peptide synthesis technology, their high-scale GMP production for clinical applications can be considered reproducible and affordable, in line with other medical device production costs [70]. We introduced bioactive scaffolds to enhance stem cell transplantation for spinal cord injury regeneration. To this aim, this study further characterized fiber morphology and the secondary structure content influenced by crosslinking treatment, presence of additives, and electrospinning synthesis process, using a multi-technique approach. Micrographs indicated the positive impact of adding HYDROSAPgp and SDS, as a reinforcer and an anionic surfactant, respectively, to modify the fibers morphology and spinnability of the electrospun peptides, resulting in uniform and finer fibers (FAQ(LDLK)_3_gp-sds and FAQ(LDLK)_3_gp-HYDROSAPgp). With regards to secondary structure proportions, IR spectroscopy indicated that crosslinking treatment, proved by multi-analysis, does not impact secondary structure content significantly. In contrast, adding HYDROSAPgp and the electrospinning process showed significant differences with regards to increasing β-sheet and α-helix, respectively. On the other hand, investigation of electrospinning solution revealed a higher proportion of β-turn and α-helix due to the influence of solvent and cosolvent on peptides’ secondary structures. With regards to cell proliferation, viability, and differentiation, hNSCs, and mNSCs, seeded on four classes of electrospun nanofibrous mats using SDS and crosslinked with Genipin, demonstrated satisfactory performances in comparison with a gold standard. It was observed to have appropriate proliferation and viability despite negligible cytotoxicity. Concerning differentiated progeny, in vitro results in 2D conditions demonstrated satisfactory cell differentiation into the three main neural phenotypes, as well as remarkable cell sprouting. The in vitro study of hNSC progeny in a 3D microchannel condition showed spread and branched morphologies with differentiated neural cells at a different stage of maturation, along with an excellent distribution of differentiated hNSCs in the whole longitudinal section. Lastly, all obtained results, supplemented by satisfactory results of foreign body reaction to implanted fibrous channels in vivo in rat spinal cords, can pave the way to the feasibility of tailored SAP microchannels as biomimetic fibrous neural conduits for spinal cord injury regeneration.

## Figures and Tables

**Table 1:** Electrospinning solutions.

Samples	Concentration (%)	FAQ(LDLK)_3_gp (%)	HYDROSAPgp (%)	SDS (%)	HFIP (%)	TFA (%)
FAQ(LDLK)_3_gp	37	100	0	0	99	1
FAQ(LDLK)_3_gp-sds	37	99	0	1	99	1
FAQ(LDLK)_3_gp-HYDROSAP	37	90	10	0	99	1
FAQ(LDLK)_3_gp-HYDROSAP-sds	37	89	10	1	99	1

## Data Availability

All data associated with the study have not been deposited in a public repository but are available from the lead contact upon reasonable request.

## Supplementary Materials

The following supporting information can be downloaded at: https://www.mdpi.com/article/10.3390/pharmaceutics15092261/s1, Figure S1: Deconvolution and second-derivative procedures were used to facilitate finding the peak positions of the amide I bands in their IR spectrums and quantitative analysis of the secondary structure components; Figure S2: Proliferation, viability, and differentiation assays of mNSCs seeded on 2D scaffolds of FAQ(LDLK)3gp, FAQ(LDLK)3gp-sds, FAQ(LDLK)3gp-HYDROSAPgp and FAQ(LDLK)3gp-HYDROSAPgp-sds for 7 days in vitro. Cultrex was used as a positive control.

## Abbreviations

SAPs	Self-assembling peptides
ECM	Extra cellular matrix
hNSCs	human neural stem cells
mNSC	murine neural stem cells
FTIR	Fourier transform infrared
SEM	Scanning electron microscope
SDS	Sodium dodecyl sulfate
HFIP	Hexafluoroisopropanol
TFA	Trifluoroacetic acid
DMF	Dimethyl formamide
EtOH	Ethanol
HCl	Hydrochloric acid
PBS	Phosphate-buffered saline
Oxyma	ethyl 2-cyano-2-(hydroxymino)acetate
DIC	N,N′-Diisopropylcarbodiimide

## References

[1] Wang T., Nanda S.S., Papaefthymiou G.C., Yi D.K. (2020). Mechanophysical Cues in Extracellular Matrix Regulation of Cell Behavior. ChemBioChem.

[2] Kusindarta D.L., Wihadmadyatami H. (2018). The role of extracellular matrix in tissue regeneration. Tissue Regeneration.

[3] Onuwaje I., Phillips J.B. (2020). Three-dimensional culture systems in central nervous system research. Handbook of Innovations in Central Nervous System Regenerative Medicine.

[4] Kharbikar B.N., Mohindra P., Desai T.A. (2022). Biomaterials to enhance stem cell transplantation. Cell Stem Cell.

[5] de Freria C.M., Van Niekerk E., Blesch A., Lu P. (2021). Neural Stem Cells: Promoting Axonal Regeneration and Spinal Cord Connectivity. Cells.

[6] Ashammakhi N., Kim H.-J., Ehsanipour A., Bierman R.D., Kaarela O., Xue C., Khademhosseini A., Seidlits S.K. (2019). Regenerative Therapies for Spinal Cord Injury. Tissue Eng. Part B Rev..

[7] Wagner F.B., Mignardot J.-B., Le Goff-Mignardot C.G., Demesmaeker R., Komi S., Capogrosso M., Rowald A., Seáñez I., Caban M., Pirondini E. (2018). Targeted neurotechnology restores walking in humans with spinal cord injury. Nature.

[8] Lewis D. (2022). Electrical stimulation helps paralysed people walk again—And now we know why. Nature.

[9] Kathe C., Skinnider M.A., Hutson T.H., Regazzi N., Gautier M., Demesmaeker R., Komi S., Ceto S., James N.D., Cho N. (2022). The neurons that restore walking after paralysis. Nature.

[10] Rosenzweig E.S., Brock J.H., Lu P., Kumamaru H., Salegio E.A., Kadoya K., Weber J.L., Liang J.J., Moseanko R., Hawbecker S. (2018). Restorative effects of human neural stem cell grafts on the primate spinal cord. Nat. Med..

[11] Kadoya K., Lu P., Nguyen K., Lee-Kubli C., Kumamaru H., Yao L., Knackert J., Poplawski G., Dulin J.N., Strobl H. (2016). Spinal cord reconstitution with homologous neural grafts enables robust corticospinal regeneration. Nat. Med..

[12] Lu P., Wang Y., Graham L., McHale K., Gao M., Wu D., Brock J., Blesch A., Rosenzweig E.S., Havton L.A. (2012). Long-Distance Growth and Connectivity of Neural Stem Cells after Severe Spinal Cord Injury. Cell.

[13] Pugliese R., Maleki M., Zuckermann R.N., Gelain F. (2019). Self-assembling peptides cross-linked with genipin: Resilient hydrogels and self-standing electrospun scaffolds for tissue engineering applications. Biomater. Sci..

[14] Zhao X., Cui K., Li Z. (2019). The role of biomaterials in stem cell-based regenerative medicine. Futur. Med. Chem..

[15] Gattazzo F., Urciuolo A., Bonaldo P. (2014). Extracellular matrix: A dynamic microenvironment for stem cell niche. Biochim. Biophys. Acta (BBA)—Gen. Subj..

[16] Adu-Berchie K., Mooney D.J. (2020). Biomaterials as Local Niches for Immunomodulation. Acc. Chem. Res..

[17] Raspa A., Carminati L., Pugliese R., Fontana F., Gelain F. (2021). Self-assembling peptide hydrogels for the stabilization and sustained release of active Chondroitinase ABC in vitro and in spinal cord injuries. J. Control. Release.

[18] Mitrousis N., Fokina A., Shoichet M.S. (2018). Biomaterials for cell transplantation. Nat. Rev. Mater..

[19] Kapałczyńska M., Kolenda T., Przybyła W., Zajączkowska M., Teresiak A., Filas V., Ibbs M., Bliźniak R., Łuczewski L., Lamperska K. (2016). 2D and 3D cell cultures—A comparison of different types of cancer cell cultures. Arch. Med. Sci..

[20] Jensen C., Teng Y. (2020). Is It Time to Start Transitioning from 2D to 3D Cell Culture?. Front. Mol. Biosci..

[21] de Lima G.G., Lyons S., Devine D.M., Nugent M.J.D. (2018). Electrospinning of Hydrogels for Biomedical Applications.

[22] Cembran A., Bruggeman K.F., Williams R.J., Parish C.L., Nisbet D.R. (2020). Biomimetic Materials and Their Utility in Modeling the 3-Dimensional Neural Environment. iScience.

[23] Gelain F., Luo Z., Zhang S. (2020). Self-Assembling Peptide EAK16 and RADA16 Nanofiber Scaffold Hydrogel. Chem. Rev..

[24] Chen J., Zou X. (2019). Self-assemble peptide biomaterials and their biomedical applications. Bioact. Mater..

[25] Mendes A.C., Stephansen K., Chronakis I.S. (2017). Electrospinning of food proteins and polysaccharides. Food Hydrocoll..

[26] Nivison-Smith L., Rnjak J., Weiss A.S. (2010). Synthetic human elastin microfibers: Stable cross-linked tropoelastin and cell interactive constructs for tissue engineering applications. Acta Biomater..

[27] Maleki M., Natalello A., Pugliese R., Gelain F. (2017). Fabrication of nanofibrous electrospun scaffolds from a heterogeneous library of co- and self-assembling peptides. Acta Biomater..

[28] Khadka D.B., Haynie D.T. (2012). Protein- and peptide-based electrospun nanofibers in medical biomaterials. Nanomed. Nanotechnol. Biol. Med..

[29] Kim K., Kloxin C.J., Saven J.G., Pochan D.J. (2021). Nanofibers Produced by Electrospinning of Ultrarigid Polymer Rods Made from Designed Peptide Bundlemers. ACS Appl. Mater. Interfaces.

[30] Hamedani Y., Macha P., Evangelista E.L., Sammeta V.R., Chalivendra V., Rasapalli S., Vasudev M.C. (2020). Electrospinning of tyrosine-based oligopeptides: Self-assembly or forced assembly?. J. Biomed. Mater. Res. Part A.

[31] Bucci R., Georgilis E., Bittner A.M., Gelmi M.L., Clerici F. (2021). Peptide-Based Electrospun Fibers: Current Status and Emerging Developments. Nanomaterials.

[32] Nuansing W., Frauchiger D., Huth F., Rebollo A., Hillenbrand R., Bittner A.M. (2013). Electrospinning of peptide and protein fibres: Approaching the molecular scale. Faraday Discuss..

[33] Pugliese R., Fontana F., Marchini A., Gelain F. (2018). Branched peptides integrate into self-assembled nanostructures and enhance biomechanics of peptidic hydrogels. Acta Biomater..

[34] Marchini A., Raspa A., Pugliese R., El Malek M.A., Pastori V., Lecchi M., Vescovi A.L., Gelain F. (2019). Multifunctionalized hydrogels foster hNSC maturation in 3D cultures and neural regeneration in spinal cord injuries. Proc. Natl. Acad. Sci. USA.

[35] Zhang K., Qian Y., Wang H., Fan L., Huang C., Yin A., Mo X. (2010). Genipin-crosslinked silk fibroin/hydroxybutyl chitosan nanofibrous scaffolds for tissue-engineering application. J. Biomed. Mater. Res. Part A.

[36] Zeng S., Ye M., Qiu J., Fang W., Rong M., Guo Z., Gao W. (2015). Preparation and characterization of genipin-cross-linked silk fibroin/chitosan sustained-release microspheres. Drug Des. Dev. Ther..

[37] Sadat A., Joye I.J. (2020). Peak Fitting Applied to Fourier Transform Infrared and Raman Spectroscopic Analysis of Proteins. Appl. Sci..

[38] Raspa A., Saracino G.A.A., Pugliese R., Silva D., Cigognini D., Vescovi A., Gelain F. (2014). Complementary Co-assembling Peptides: From In Silico Studies to In Vivo Application. Adv. Funct. Mater..

[39] Marchini A., Favoino C., Gelain F. (2020). Multi-Functionalized Self-Assembling Peptides as Reproducible 3D Cell Culture Systems Enabling Differentiation and Survival of Various Human Neural Stem Cell Lines. Front. Neurosci..

[40] Butler M.F., Ng Y.-F., Pudney P.D.A. (2003). Mechanism and kinetics of the crosslinking reaction between biopolymers containing primary amine groups and genipin. J. Polym. Sci. Part A Polym. Chem..

[41] Roy S., Rhim J.-W. (2022). Genipin-Crosslinked Gelatin/Chitosan-Based Functional Films Incorporated with Rosemary Essential Oil and Quercetin. Materials.

[42] Friedman M. (2004). Applications of the Ninhydrin Reaction for Analysis of Amino Acids, Peptides, and Proteins to Agricultural and Biomedical Sciences. J. Agric. Food Chem..

[43] Zatorski J.M., Montalbine A.N., Ortiz-Cárdenas J.E., Pompano R.R. (2020). Quantification of fractional and absolute functionalization of gelatin hydrogels by optimized ninhydrin assay and ^1^H NMR. Anal. Bioanal. Chem..

[44] Zhang Y., Wang Q.-S., Yan K., Qi Y., Wang G.-F., Cui Y.-L. (2016). Preparation, characterization, and evaluation of genipin crosslinked chitosan/gelatin three-dimensional scaffolds for liver tissue engineering applications. J. Biomed. Mater. Res. Part A.

[45] Di Foggia M., Taddei P., Torreggiani A., Dettin M., Tinti A. (2012). Self-Assembling Peptides for Biomedical Applications: IR and Raman Spectroscopies for the Study of Secondary Structure. Proteom. Res. J..

[46] Barth A. (2007). Infrared spectroscopy of proteins. Biochim. Biophys. Acta Bioenerg..

[47] Kong J., Yu S. (2007). Fourier Transform Infrared Spectroscopic Analysis of Protein Secondary Structures. Acta Biochim. Biophys. Sin..

[48] Mazzini L., Gelati M., Profico D.C., Sorarù G., Ferrari D., Copetti M., Muzi G., Ricciolini C., Carletti S., Giorgi C. (2019). Results from Phase I Clinical Trial with Intraspinal Injection of Neural Stem Cells in Amyotrophic Lateral Sclerosis: A Long-Term Outcome. Stem Cells Transl. Med..

[49] Forouharshad M., King S.G., Buxton W., Kunovski P., Stolojan V. (2019). Textile-Compatible, Electroactive Polyvinylidene Fluoride Electrospun Mats for Energy Harvesting. Macromol. Chem. Phys..

[50] Zheng J.-Y., Zhuang M.-F., Yu Z.-J., Zheng G.-F., Zhao Y., Wang H., Sun D.-H. (2014). The Effect of Surfactants on the Diameter and Morphology of Electrospun Ultrafine Nanofiber. J. Nanomater..

[51] Gardella L., Forouharshad M., Pastorino L., Monticelli O. (2017). Hyperbranched PDLA-polyglicerol: A novel additive for tuning PLLA electrospun fiber degradation and properties. Eur. Polym. J..

[52] Han S., Nie K., Li J., Sun Q., Wang X., Li X., Li Q. (2021). 3D Electrospun Nanofiber-Based Scaffolds: From Preparations and Properties to Tissue Regeneration Applications. Stem Cells Int..

[53] Gelain F., Cigognini D., Caprini A., Silva D., Colleoni B., Donegá M., Antonini S., Cohen B.E., Vescovi A. (2012). New bioactive motifs and their use in functionalized self-assembling peptides for NSC differentiation and neural tissue engineering. Nanoscale.

[54] Pugliese R., Marchini A., Saracino G.A.A., Zuckermann R.N., Gelain F. (2018). Cross-linked self-assembling peptide scaffolds. Nano Res..

[55] Tian L., Prabhakaran M.P., Ramakrishna S. (2015). Strategies for regeneration of components of nervous system: Scaffolds, cells and biomolecules. Regen. Biomater..

[56] Behtaj S., John J.A.S., Ekberg J.A.K., Rybachuk M. (2022). Neuron-fibrous scaffold interfaces in the peripheral nervous system: A perspective on the structural requirements. Neural Regen. Res..

[57] Anderson J.M., Rodriguez A., Chang D.T. (2008). Foreign body reaction to biomaterials. Semin. Immunol..

[58] Westerman M., Spencer J., Collet C. (1991). Chromosomal localization of the gene for late lactation protein (LLP) in the tammar wallaby (*Macropus eugenii*). Cytogenet. Genome Res..

[59] Morais J.M., Papadimitrakopoulos F., Burgess D.J. (2010). Biomaterials/Tissue Interactions: Possible Solutions to Overcome Foreign Body Response. AAPS J..

[60] de Jong E.K., Dijkstra I.M., Hensens M., Brouwer N., van Amerongen M., Liem R.S.B., Boddeke H.W.G.M., Biber K. (2005). Vesicle-Mediated Transport and Release of CCL21 in Endangered Neurons: A Possible Explanation for Microglia Activation Remote from a Primary Lesion. J. Neurosci..

[61] Perale G., Rossi F., Sundstrom E., Bacchiega S., Masi M., Forloni G., Veglianese P. (2011). Hydrogels in Spinal Cord Injury Repair Strategies. ACS Chem. Neurosci..

[62] Batchelor P.E., Liberatore G.T., Wong J.Y.F., Porritt M.J., Frerichs F., Donnan G.A., Howells D.W. (1999). Activated Macrophages and Microglia Induce Dopaminergic Sprouting in the Injured Striatum and Express Brain-Derived Neurotrophic Factor and Glial Cell Line-Derived Neurotrophic Factor. J. Neurosci..

[63] Nagamoto-Combs K., Morecraft R.J., Darling W.G., Combs C.K., Orihuela R., McPherson C.A., Harry G.J., Manocha G.D., Puig K., Spejo A.B. (2010). Long-Term Gliosis and Molecular Changes in the Cervical Spinal Cord of the Rhesus Monkey after Traumatic Brain Injury. J. Neurotrauma.

[64] Okada S., Hara M., Kobayakawa K., Matsumoto Y., Nakashima Y. (2018). Astrocyte reactivity and astrogliosis after spinal cord injury. Neurosci. Res..

[65] Lukovic D., Stojkovic M., Moreno-Manzano V., Jendelova P., Sykova E., Bhattacharya S.S., Erceg S. (2015). Concise Review: Reactive Astrocytes and Stem Cells in Spinal Cord Injury: Good Guys or Bad Guys?. Stem Cells.

[66] Low W.C., Rujitanaroj P.O., Lee D.K., Messersmith P.B., Stanton L.W., Goh E., Chew S.Y. (2013). Nanofibrous scaffold-mediated REST knockdown to enhance neuronal differentiation of stem cells. Biomaterials.

[67] Raspa A., Gelain F. (2021). Mimicking Extracellular Matrix via Engineered Nanostructured Biomaterials for Neural Repair. Curr. Neuropharmacol..

[68] Fang A., Li D., Hao Z., Wang L., Pan B., Gao L., Qu X., He J. (2019). Effects of astrocyte on neuronal outgrowth in a layered 3D structure. Biomed. Eng. Online.

[69] Hu Y., Huang G., Tian J., Qiu J., Jia Y., Feng D., Wei Z., Li S., Xu F. (2021). Matrix stiffness changes affect astrocyte phenotype in an in vitro injury model. NPG Asia Mater..

[70] Gelain F., Luo Z., Rioult M., Zhang S. (2021). Self-assembling peptide scaffolds in the clinic. NPJ Regen. Med..

