# Supplementary material for: Biomimetic Electrospun Self-Assembling Peptide Scaffolds for Neural Stem Cell Transplantation in Neural Tissue Engineering
Source: Pharmaceutics. 2023 Aug 31;15(9):2261. doi: 10.3390/pharmaceutics15092261 (PMC10536048; doi:10.3390/pharmaceutics15092261)
Supplement: Supplementary file 1 [file pharmaceutics-15-02261-s001.zip › pharmaceutics-2556820-supplementary.pdf]

Biomimetic Electrospun Self-Assembling Peptide Scaffolds for Neural Stem Cell Transplantation in Neural Tissue Engineering

Mahdi Forouharshad[1], Andrea Raspa[1,2], Amanda Marchini[1], Maria Gessica Ciulla[1], Alice Magnoni[3], Fabrizio Gelain[1,2,*]

[1]Institute for Stem-Cell Biology, Regenerative Medicine and Innovative Therapies, IRCCS Casa Sollievo della Sofferenza, 71013 San Giovanni Rotondo, Italy
[2]Center for Nanomedicine and Tissue Engineering (CNTE), ASST Grande Ospedale Metropolitano Niguarda, 20162 Milan, Italy
[3]Department of Biotechnology and Biosciences, University of Milan – Bicocca, Piazza della Scienza 2, 20125 Milan, Italy.

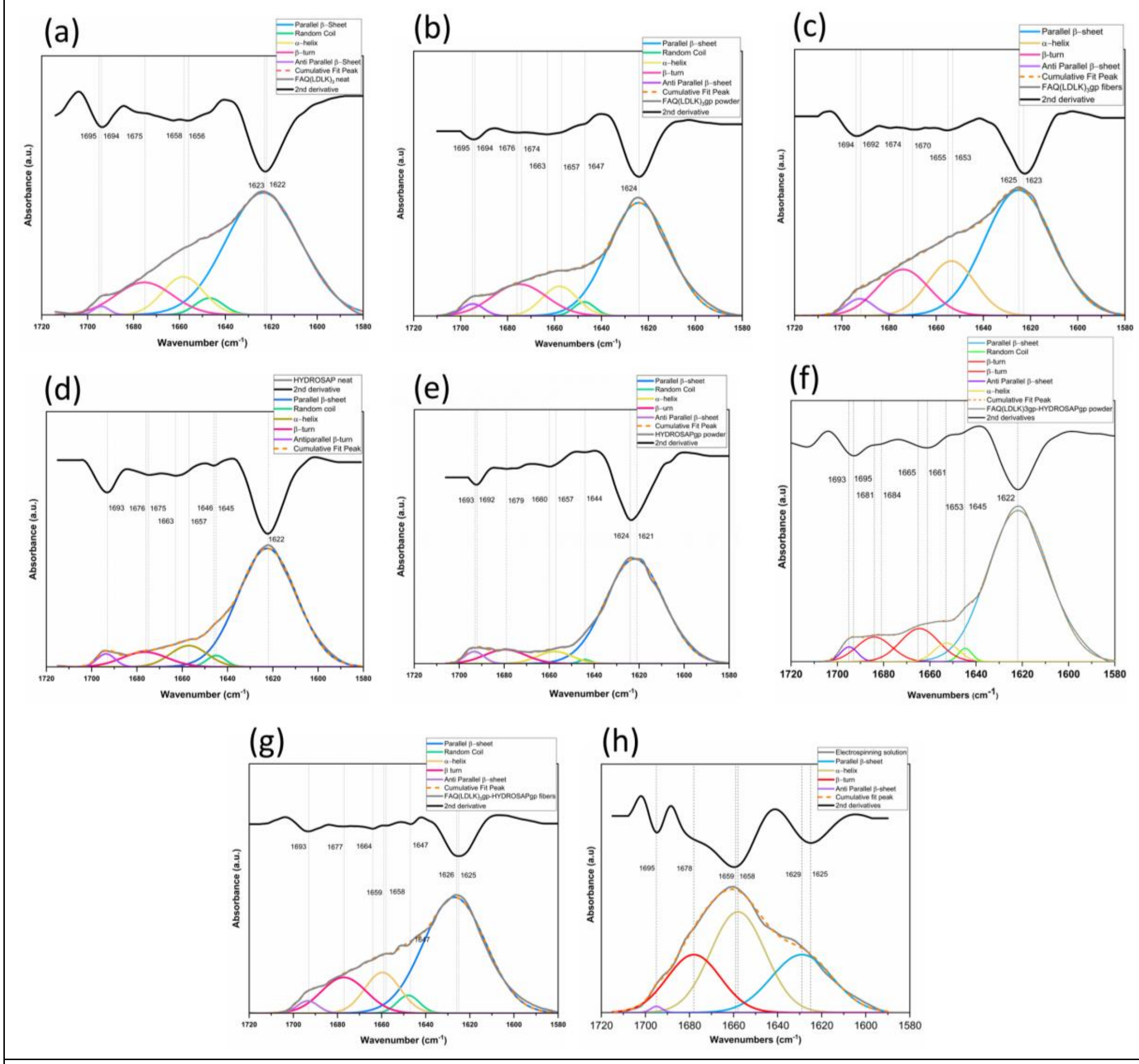

Figure S1. Deconvolution and second-derivative procedures were used to facilitate finding the peak positions of the amide I bands in their IR spectrums and quantitative analysis of the secondary structure components. (a) FAQ(LDLK)$_3$ neat, (b) FAQ(LDLK)$_3$gp powder, (c) FAQ(LDLK)$_3$gp fibers, (d) HYDROSAP neat, (e) HYDROSAPgp powder, and (f) FAQ(LDLK)$_3$gp-HYDROSAPgp powder, (g) FAQ(LDLK)$_3$gp-HYDROSAPgp fibers, and (h) electrospinning solution.

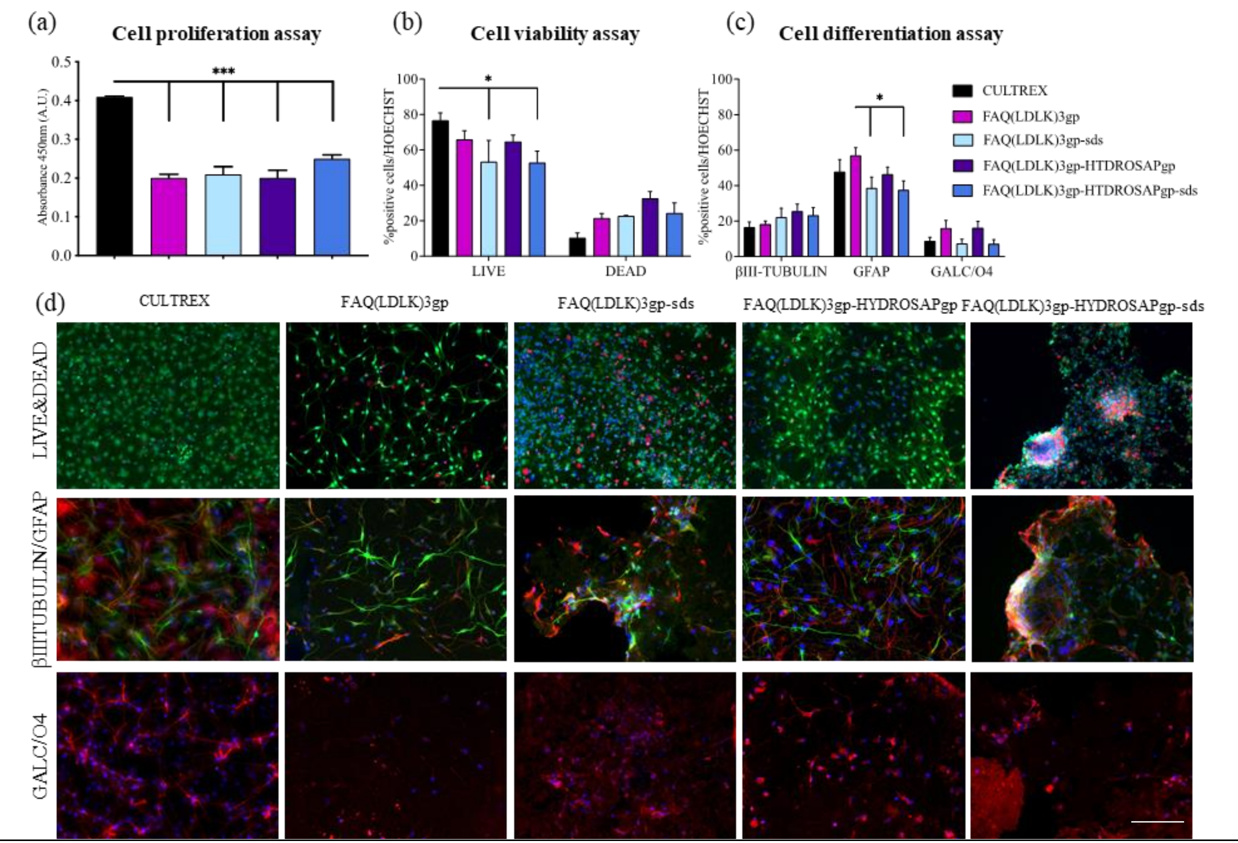

Figure S2. Proliferation, viability, and differentiation assays of mNSCs seeded on 2D scaffolds of FAQ(LDLK)3gp, FAQ(LDLK)3gp-sds, FAQ(LDLK)3gp-HYDROSAPgp and FAQ(LDLK)3gp-HYDROSAPgp -sds for 7 days in vitro. Cultrex was used as a positive control. (a) Colorimetric MTS assay for measurement of cell proliferation. Absorbance values were detected at 490 nm (A.U., Arbitrary Units). (b) LIVE/DEAD Cell Viability/Cytotoxicity test to determine cell viability. (c) Quantification of positive cells for βIII-Tubulin (neurons), GFAP (astrocytes), and GALC/O4 (oligodendrocytes). (d) Representative fluorescence images for cell viability assay (top), neural and astroglial differentiation (middle), and oligodendroglial differentiation (bottom). Live cells are labeled in green, dead cells in red; neurons are stained with the βIII-tubulin marker in green, astrocytes with GFAP marker in red, and oligodendrocytes in red with GALC/O4 marker. Cell nuclei were stained with HOECHST (in blue). Data are represented as mean ±SEM. Statistical analysis shows significant differences between conditions ($^*p < 0.05$; $^{***}p < 0.001$). All measures were performed in triplicate. Scale bar, 100 µm.
